# Cost data in implementation science: categories and approaches to costing

**DOI:** 10.1186/s13012-021-01172-6

**Published:** 2022-01-28

**Authors:** Heather T. Gold, Cara McDermott, Ties Hoomans, Todd H. Wagner

**Affiliations:** 1 NYU Langone Health, 550 First Ave, VZ30, Sixth Floor, New York, NY 10016 USA; 2 University of Washington, Seattle, WA USA; 3 London School of Economics and Political Science, London, UK; 4 Palo Alto VA and Stanford University, Palo Alto, CA USA

**Keywords:** Implementation costs, Intervention costs, Cost analysis, Implementation economics

## Abstract

A lack of cost information has been cited as a barrier to implementation and a limitation of implementation research. This paper explains how implementation researchers might optimize their measurement and inclusion of costs, building on traditional economic evaluations comparing costs and effectiveness of health interventions. The objective of all economic evaluation is to inform decision-making for resource allocation and to measure costs that reflect opportunity costs—the value of resource inputs in their next best alternative use, which generally vary by decision-maker perspective(s) and time horizon(s). Analyses that examine different perspectives or time horizons must consider cost estimation accuracy, because over longer time horizons, all costs are variable; however, with shorter time horizons and narrower perspectives, one must differentiate the fixed and variable costs, with fixed costs generally excluded from the evaluation. This paper defines relevant costs, identifies sources of cost data, and discusses cost relevance to potential decision-makers contemplating or implementing evidence-based interventions. Costs may come from the healthcare sector, informal healthcare sector, patient, participant or caregiver, and other sectors such as housing, criminal justice, social services, and education. Finally, we define and consider the relevance of costs by phase of implementation and time horizon, including pre-implementation and planning, implementation, intervention, downstream, and adaptation, and through replication, sustainment, de-implementation, or spread.

Contributions to the literature

- A lack of cost information has been cited as a barrier to implementation and a limitation of implementation research; therefore, this paper explains how implementation researchers might optimize their measurement and inclusion of costs, building on traditional economic evaluations of health interventions.
- The paper bridges health economics and implementation science to guide researchers in their approaches to costing in implementation studies.
- This paper defines and considers the relevance of costs by phase of implementation and time horizon, including pre-implementation and planning, implementation, intervention, downstream, and adaptation, and through replication, sustainment, de-implementation, or spread.

## Background

Costs have been identified as a key outcome in implementation research [1, 2], yet there is little guidance on how to measure costs as distinct from more traditional cost-effectiveness analyses. Importantly, an implementation strategy may affect its target intervention and have further downstream impacts [2], all of which should be considered in the analysis [3]. Economic evaluations (comparing alternatives based on both costs and benefits) can inform resource allocation decisions and influence stakeholder acceptability, adoption, or sustainability of an implementation strategy or intervention [4–6]. By measuring costs, implementation scientists can answer questions ranging from what are the costs of alternative implementation strategies in a particular context to what would it cost to replicate these strategies elsewhere. In this paper, we identify costs relevant to economic evaluation in implementation science and discuss practical approaches to costing—paying particular attention to such aspects of evaluation as a decision-maker’s objectives, perspectives, and time horizon.

## Types of costs to consider measuring for an implementation study

**Fig. 1:**
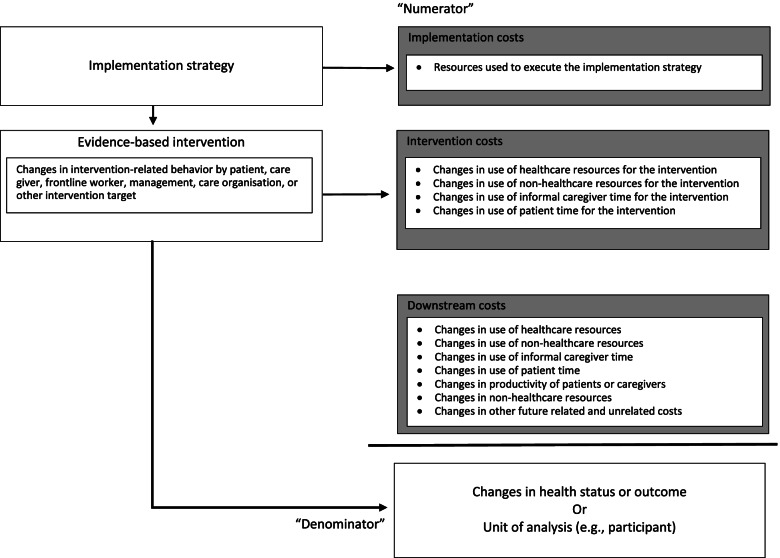
Economic consequences of implementing health interventions

Costs are incurred when employing an implementation strategy [7]. If the strategy is effective at changing the use of its targeted evidence-based intervention, then this leads to a change in intervention costs. The strategy can also lead to intended and unintended downstream effects and costs, as Fig. 1 illustrates. These distinctions in costs—implementation, intervention, and downstream—are different from costs by implementation phase, described in the section “Costs by implementation phase,” and may include broad representation across types of costs, such as healthcare, patient, caregiver, productivity, or other sectors. The cost categories described here serve as a guide to determine which costs may be important to capture, relative to the research question under consideration.

### Implementation costs

Implementation costs are those related to the development and execution of the implementation strategy that targets one or more specific evidence-based interventions. The strategy will affect directly the subsequent intervention, possibly in its efficiency, utilization, or quality. For example, a recent US study examining the costs of implementing collaborative care to improve depression treatment tracked the costs of recruiting and employing case managers to coordinate services across organizations and staff; costs of training psychiatric consultants, social workers, and other care staff; costs of installing information technology; and costs accrued to study sites for planning local strategies to implement collaborative care [8]. The Expert Recommendations for Implementing Change [7] highlight many potential implementation strategies, which may operate separately or in conjunction with each other and which may differ in resource consumption and costs. The cost of implementation strategies can be assessed as a total, and they can also be captured by unique components or implementation phase [9]. This is discussed further in the section “Costs by implementation phase.”

Implementation strategies may impose efficiency losses or gains when clinicians and other frontline workers participate in educational activities or training (instead of providing or “producing” direct care or services). There are two approaches to consider for these costs. One is to collect primary data to measure these activities. Another approach is to assume that the added time is minimal and can be ignored. The latter approach is appealing for its simplicity, but results can be misleading if these costs are greater than zero. For example, a program might assume that the implementation effort will be handled by primary care providers, who in a typical day spend 27% of their time face-to-face with patients and about 1–2 h nightly on documentation [10]. Consequently, assuming that the additional 10–15 min of implementation activity is unimportant may bias costing and result in implementing a burdensome and infeasible intervention from the start. Another example is of healthcare systems adapting and utilizing telehealth interventions, which may add a cost of technology for both providers and patients [11]. There may be some substitution of labor and capital for such telehealth services, yet there may be additional costs or savings, too.

### Intervention costs

These are resource costs that result as a direct consequence of implementation strategies targeting evidence-based interventions. For example, implementation scientists may seek to increase the use of tissue plasminogen activator (tPA) for patients with ischemic stroke. In this case, the intervention costs are those associated with specific changes in the use of tPA. The intervention costs increase with uptake, whereas de-implementation studies typically seek to reduce the use of an intervention and its related costs. Intervention costs should also consider the costs borne by patients, participants, or caregivers, as a result of the intervention. In addition, the intervention may require other types of resources such as non-healthcare resources (e.g., transportation or childcare costs), informal care assistance and time, and patient resource costs (including time). Whenever possible, researchers should estimate the intervention costs separately from the implementation costs [12].

### Downstream costs

We define downstream costs as those that are further subsequent to the implemented intervention and may have changed, intentionally or unintentionally, as a result of the implementation strategy and intervention. Potential downstream costs include healthcare utilization and costs, productivity costs of patients and caregivers, costs in sectors other than healthcare, and future related and unrelated health and non-health costs that accrue to the initial decision-maker or to other stakeholders. As shown in Fig. 1, both intervention and downstream costs can share the same cost categories, such as healthcare costs. Hence, it is important not to double count the same costs in both categories.

Our definition of downstream costs is different from traditional clinical cost-effectiveness analytic methods [13, 14]. We suggest separating implementation strategy and intervention costs from subsequent downstream cost impacts of the intervention. Many implementation studies measure the implementation and intervention costs, but not further downstream costs. This is sometimes justified because prior research showed that increasing intervention use was cost-effective, with effective implementation leading to improved health outcomes that extend life. Future costs reflect resources associated with future changes that arise from an intervention and its implementation. For example, a seemingly simple prevention effort such as an implementation trial to improve the use of nicotine replacement therapy for smoking cessation might center on whether the implementation trial increased the costs of delivering nicotine replacement therapy, assuming that the downstream benefits of smoking cessation are known. There is a danger, however, in assuming the intervention is cost-effective, because by adding implementation costs, in many cases, the cost-effectiveness of the intervention could decrease [15]. Incorporating future costs and savings into the analysis may counterbalance the implementation costs based on subsequent reduction in lung and heart disease. Any implementation strategy and intervention could lead to intended and unintended consequences in the near and longer term that should be measured. When enumerating intervention and downstream costs, it is possible to include the identical costs or cost components in multiple categories. However, this should be avoided because it can lead to double counting and a biased, overestimated total cost.

Several additional factors should be considered when deciding whether or not to include downstream costs: (1) organizational complexity of the implementation strategy and intervention; (2) number of people affected by the intervention; (3) time horizon and ability to capture downstream costs and effects; (4) whether the intervention focuses on more acute issues versus preventive services with a late, long-term impact; and (5) stakeholder perspective. Unless there is a strong justification not to do so, we recommend tracking downstream costs, especially because these costs—intended and unintended—could affect sustainability and penetration, and may be of interest to various stakeholders.

Besides the distinction between implementation, intervention, and downstream costs, there are different cost categories to consider that depend importantly on the study’s stakeholders, analytic perspective, and time horizon—evaluation aspects on which we expand later and that inform the decisions about which costs to capture. A brief discussion of the different cost categories includes healthcare costs, patient costs, caregiver costs, productivity costs, other sector costs, future costs, and the typically excluded research and development costs.

- Healthcare costs
  The costs of healthcare resources could come from hospitals (i.e., inpatient and outpatient care costs), laboratory services and procedures, imaging tests, clinician services (e.g., from physicians, nurses, or other providers such as social work, psychologists, or dentists), medications, hospice care, home care, nursing homes, and other health service and education providers (e.g., community health workers, practice facilitators). Typically, healthcare utilization and costs are measured at the patient level (see Table 1) [16]. These costs may be higher or lower than previous estimates, based on how well the implementation strategy stimulates utilization of the intervention and subsequent healthcare services. If the rollout of the intervention is inefficient, for example, potentially more healthcare services could be required to compensate for a poor rollout, thereby increasing healthcare costs; alternatively, a highly efficient implementation could reduce healthcare service use and reduce overall healthcare costs. As we discuss in more detail below, implementation researchers should focus on measuring the cost of producing healthcare, that is, the actual cost of inputs such as clinician time, medical supplies, and equipment; measuring charges, prices, or payments may provide misleading cost estimates, because they may be considerably different from the actual cost of producing healthcare [17]. Healthcare should include resources used, intentionally or unintentionally, due to the implementation strategy and intervention. For example, strategies to increase the use of tPA to treat patients with stroke might result in a decrease in skilled nursing facility and stroke rehabilitation care (intentional) and an increase in further acute medical care (unintentional).

**Table 1:** Cost impact inventory in implementation science

Cost category	Type of impact	Analytic perspective						Phase^a^
		Societal	Healthcare sector	Criminal Justice	Patient/caregiver	Public health dept	Other^b^	
**Implementation strategy (or de-implementation)**	Paid by stakeholder/implementer. Costs include intervention adaptation and planning for implementation	x	x (only if implemented by the healthcare sector)		n/a	X (only if implemented by public health dept)		i–ii
**Formal healthcare sector** **medical costs**	1. Paid for by third-party payers	x (all)	x (all)					iii–iv iii–iv
	2. Paid for by patients out-of-pocket				x			iv
	3. Future related medical costs (payers and patients) 4. Future unrelated medical costs (payers and patients)				x			iv
**Informal healthcare sector**	1. Patient-time costs	x (all)	n/a	n/a	x			ii–iii
	2. Unpaid caregiver-time costs				x			
	3. Transportation costs				x			
**Non-healthcare sectors**		x (all)	n/a					
**Productivity**	1. Labor market earnings lost		n/a		x			
	2. Cost of unpaid lost productivity due to illness				x			
	3. Cost of uncompensated household production				x			
**Consumption**	Future consumption unrelated to health		n/a					iv
**Social services**	1. Cost of social services as part of the intervention or implementation strategy		n/a			x (possibly)	x (possibly)	i–ii
	2. Cost/savings of social services downstream							iii–iv
**Legal or criminal justice**	1. Number and cost of crimes related to intervention		n/a	x				iii
	2. Cost of crimes downstream			x				iv
**Education**	Impact of intervention on the educational achievement of population		n/a				x (possibly)	iv
**Housing**	Cost of intervention on home improvements (e.g., removing lead paint)		n/a				x (possibly)	ii–v
**Environment**	Production of toxic waste pollution by intervention		n/a				x (possibly)	iv–v
**Others (specify)**	Other impacts		n/a					

Adapted from Sanders/Neumann [13, 16]

^a^Phases include:

i.Exploration/pre-implementation/preparation costs, sometimes includes adaptation

ii.Implementation costs

iii.Intervention costs

iv.Downstream costs

v.Sustainment, adaptation, spread, de-implementation

^b^Analytic perspectives could include societal, patient, caregiver, healthcare system, healthcare sector, justice system, public health department, housing (public, private), environmental sector, among others. When substitution of costs across sectors is expected by intervention, costs from any involved sectors should be counted
- Patient costs
  Patient costs include healthcare-related out-of-pocket costs (e.g., copayments, coinsurance); time required for treatment, waiting, and travel; transportation costs (e.g., public transportation fare, parking, automobile mileage); and dependent-care costs (e.g., day care) during treatment of the participant or patient. For instance, a nutrition intervention may require patients to incur cost of dietary changes. Another example is the time required for patients receiving oncology care [18]. Previous work describes patients’ costs and ways to value patient time [19].
- Caregiver costs
  Sometimes caregivers are paid employees, but often caregivers are family members or friends. Caregiver costs include time and transportation to, from, and during the intervention, if the caregiver is accompanying the patient or participant. This can be non-trivial, for example, for lengthy hospital stays or on-going health education programs. Caregiver costs may also include the time of home-based care of a participant or patient at the cost of what it would have been if provided by paid staff, even if time were “donated” by a family member or friend. Caregiver costs are also termed “spillover effects.” A positive spillover effect would be reduced caregiving time resulting from improved patient health following an intervention [20], whereas negative spillover would increase caregiving needs [21]. There is controversy over how to value caregiver time [16, 22]. Often analysts use the minimum wage or the average wage rate for personal aides, as a proxy for opportunity cost.
- Productivity costs
  Productivity costs refer to changes in earnings (i.e., loss or gain) of patients and caregivers due to changes in health or illness. Whether to include changes in earnings in economic evaluations has been debated, with guidance shifting over time [16, 23]. Presenteeism (being at work but not fully productive) may require primary data collection and has productivity implications, as does absenteeism [24], which can sometimes be measured in administrative data [25, 26]. Valuing changes in earnings can be controversial for older adults no longer in the workforce or people who are disabled, relative to typical working-age people [16, 23]. Furthermore, there may be productivity costs associated with caregivers leaving the workforce to provide informal patient care, which can lead to large financial burdens [27]. Using survey assessments of changes in time use can guide costing of productivity changes, although productivity changes, and therefore costs, often are overestimated and require extensive sensitivity analyses. Importantly, some patient or caregiver costs may be considered productivity costs, such as lost time from work; the analyst must ensure they do not double count these costs in the evaluation.
- Other sector costs
  Other sectors besides healthcare may be impacted by implementation activities, including criminal justice, housing, or education sectors, social services, or the environment [16]. For example, an implementation study seeking to expand the reach of methadone-assisted treatment might consider costs or savings in the criminal justice sector.
- Research and development costs
  Traditional methods in economic evaluation suggest excluding research and development costs of the implementation strategies or interventions themselves. Research-specific activities that were essential to designing and developing the original implementation generally should be excluded, sometimes called pre-implementation or design components [9, 28]. For example, we would exclude the time required for survey completion or laboratory tests that were only used for evaluating implementation study outcomes, if those surveys or laboratory tests would not be part of usual care [3, 29]. Two exceptions to this approach may be considered. The first is when the stakeholder wants to know the total cost of implementation, including these research-specific and sunk costs. The second is when there are research activities required for implementation planning or adaptation within a specific context. In both cases, these costs should be included.

## Costing: accuracy and precision

The “gold standard” for measuring costs is to estimate opportunity costs, which are defined as the value of something that is given up to acquire the next best option, or what was foregone by purchasing what was purchased. Opportunity costs are specific to the decision-maker and they may change as the time horizon changes. Market prices sometimes provide a good estimate of opportunity costs. When estimating the cost of supplies, such as computers or bandages, it is easy to observe the unit costs by searching market prices on the Internet. In other sectors, such as prisons and healthcare, market prices for estimating the average cost of a 4-day stay are typically not readily available, in which case, production costs are used as proxies for opportunity costs.

- Distinguishing fixed and variable costs
  Implementation analyses should differentiate between fixed and variable costs whenever possible. Accountants usually define fixed cost as costs for resources that do not change in a fiscal period, which might be a month or a year. Economists view fixed and variable costs based on whether they vary with scale of production (i.e., increasing use of the strategy or intervention would increase resources used). Seeing more patients requires more labor and supplies, which are variable costs that refer to relatively small changes in production. Another example is implementing additional “tweaks” to an information technology system as part of an implementation strategy (e.g., decision support), which would yield variable costs, but the information technology system itself would be considered a fixed cost. Equipment such as CT scanners, buildings, or costs for developing an educational program as an implementation strategy are fixed and do not change with scaling up, although there may be a future decision to purchase a new scanner or building when production is large enough.
  In the long term, when scale of production can see large changes in variation, all costs can be considered variable [30]. In the short run, fixed costs should be excluded from the evaluation because they create no opportunity cost. There are two challenges with this approach. First, it is often possible to convert fixed costs to variable costs. For example, a hospital can convert inpatient beds to an outpatient clinic, or it could sell the space. In doing so, the hospital is converting the fixed costs into another type of fixed cost or a variable asset. Second, many implementation scientists use shorter time horizons, or a range of time horizons, where some costs may be fixed or variable, depending on the time horizon. In analysis with very short time horizons, fixed costs cannot be converted. However, as the time horizon increases, one has more options for converting fixed costs to variable, and this creates substantial analytic complexity. New healthcare evidence and interventions often prompt organizations and practitioners to reallocate inputs that can be changed immediately and reconsider what care and services to produce from all available resources, especially in the long run. Including fixed costs inappropriately would bias the total costs upwards. Implementation analyses should differentiate between fixed and variable costs whenever possible. Typically, fixed costs should be excluded from the evaluation unless the analysis uses a time horizon where the fixed costs may be converted to variable costs.
- Accuracy
  Researchers may ask, “how accurate do cost data need to be?” Accuracy describes the extent to which cost estimates reflect the “true” opportunity cost. In some controlled studies, the same costs may be incurred in the intervention and control groups. When computing the difference in costs between the intervention and control arm, these costs cancel each other out and may be excluded. However, including these costs has two advantages. First, it allows one to check whether the costs are the same in both groups. Second, including these costs may facilitate comparisons to other studies. Whether these costs are included or excluded needs to be stated explicitly in publications so the reader is not misled. Finally, more attention should be placed on accurately measuring large and highly variable costs. For example, implementation scientists should spend more effort measuring costs for hospitalizations, which are expensive, compared with brief phone calls, all else being equal.
- Precision
  Costing may also raise questions about precision. Precision depends on the unit of measurement and how finely the resources are measured. For example, imagine a study designed to use practice facilitation to implement telerehabilitation for patients who experienced a major cardiac event. It would be relatively easy to develop a one-time survey or interview to estimate accurately the average cost of this intervention [31]. However, a one-time survey would not be very precise. A much more precise measure would require tracking the time for each encounter using time-in-motion studies [32, 33], whereas a compromise could be tracking time for a random sample of encounters. Other advances to estimating time include time-tracking software for activities (e.g., Salesforce™, phone records), mobile phone apps for measuring implementation costs, and building in reporting into the electronic health record of a healthcare system. Based on gathered time estimates, one can estimate costs by applying national estimates of the average wage and fringe rates for the relevant job categories, for example from the US Bureau of Labor Statistics [34]. Estimating costs precisely is time consuming and expensive. Additional precision is necessary when comparing close substitutes and when estimating subgroup effects [12], but otherwise, it may be unnecessary. Researchers should design their studies with consideration of these trade-offs.
- Approaches to costing
  Micro-costing has been used widely in the health economics literature. This involves tracking all of the inputs used to make a product or provide a service. Costs are estimated by multiplying the quantity of these inputs by their input costs. Many implementation studies will estimate costs using micro-costing methods, which can vary in their precision. The term micro-costing methods refers to a set of similar methods with different names, such as time and motion (TM) studies or time-driven activity-based costing (TD-ABC). Both TM and TD-ABC involve tracking the time it takes to conduct activities. Often process maps are linked to specific activities, resources, and events and their frequencies are calculated. The time for each activity is multiplied by appropriate wage rates and then summed to estimate total costs [33]. Micro-costing is most frequently used to estimate the cost of an implementation strategy.

Micro-costing is prohibitively time consuming for estimating the cost of an inpatient stay. Fortunately, some organizations track labor and supply costs using activity-based cost (ABC) accounting databases to estimate their production costs. These systems track the variable costs provided to each patient. These databases also track fixed, capital costs, including equipment and space, and hospital overhead (e.g., utilities, human resources, security). ABC databases have become the gold standard for estimating healthcare costs in economic evaluation [35]. These systems estimate the production cost and often include subtotals that can be useful for implementation researchers. While beneficial for the specific system costs, the potential limitation of ABC systems is that the production costs reflect only that specific organization or health system and all its unique attributes.

An increasing number of hospitals and healthcare systems are investing in ABC systems, because they enable the hospital managers to examine their production costs [36, 37], but there are implicit assumptions about opportunity costs built into these systems. If an implementation researcher is studying the costs related to an implementation strategy at a specific hospital, then the ABC data provide opportunity costs for that specific hospital and time frame. However, opportunity costs are specific to the decision-maker’s perspective and they change as the time horizon changes.

Because not all organizations use ABC systems, another estimation approach is to use macro-costing, also called gross costing, which is based on grouped or aggregated resources used [13, 38]. Organizations may have information on their charges for a service and its associated components (e.g., hospital stay, laboratory test), which is simply the amount listed on bills for a group of services and resources, which could be adjusted to estimate costs. Charges include profits and can be used in negotiations with third-party payers, so any charges need to be deflated to represent costs more closely, for example, by applying a hospital or hospital department’s cost-to-charge ratio [23]. Payments for services, especially federal reimbursement rates such as through the US Medicare program, provide another common source of proxy cost information. The payment is the amount paid by the insurer or public agency, and in the case of the US Medicare program, is based on work effort, overhead, and other operating costs associated with providing healthcare services. For US medication cost estimates, the US Federal Supply Schedule is recommended based on Veterans Affairs purchasing prices [22]. A limitation with both charges and payments is the inability to separate variable and fixed costs. The inclusion of fixed costs can confound an analysis with a short time horizon; not only do the fixed costs overstate the costs that can be varied, but variation in the fixed costs can also reduce the power to detect meaningful differences in variable costs [3].

There are national databases in the USA, such as the Healthcare Cost and Utilization Project (HCUP) [39], Medicare administrative claims, or commercially available health insurance claims databases, that can be analyzed to provide details on average charges or payments for services, which can be a complicated process [40–43]. Finally, any relevant out-of-pocket healthcare costs to patients can be calculated from some claims databases using copayments, coinsurance, or deductible amounts to assess patient costs and the full healthcare sector cost. However, in each case, these estimates may have little relevance to the opportunity cost of a specific hospital(s) or organization being studied, because each entity has its own way of converting inputs into patient care and outcomes.

How to generalize cost estimates from specific healthcare systems or settings is a question that remains. However, national-level estimates of costs and disaggregated resources required may be the most useful so that local organizations can adjust to their locality. At the very least, one can conduct sensitivity analysis to assess a range of costs that might be relevant to various localities (e.g., vary costs ± 5–10% or by a cost-of-living adjustment). Importantly, using data from one hospital or organization for another makes implicit assumptions about the production process. Variation in hospital efficiency may be less consequential in economic evaluations that use a long-run time horizon, but they can be important when assessing variable costs in the short run [44, 45].

Regardless of the potential difficulties, implementation scientists should focus on production costs when estimating costs, which are likely to lead to the best approximation of opportunity costs for implementation studies, with special attention to the contextual issues relevant to estimating production costs. Because there is no database reporting opportunity costs, implementation researchers might consider qualitative work prior to estimating production costs to ensure that the estimates reflect the variable cost within a decision-maker’s time horizon [4].

## Costs by implementation phase

Different types of costs may be incurred by stage in the implementation process [9] (Table 1). Those stages could include pre-implementation, exploratory, and planning, as well as adaptation, implementation, sustainment, spread, and de-implementation [46]. Summing costs by phase can be useful for stakeholders to determine the feasibility of implementation over time [28]. Costs ascertained from any of these phases should be included if they are required to replicate the implementation strategy and associated intervention elsewhere (or to adapt, sustain, spread, or de-implement the strategy and intervention). Sometimes it can be difficult to disentangle costs by phase, for example, implementation and intervention costs, or implementation and adaptation costs. It is important to note where there may be overlap or lack of clarity on phase distinction. Furthermore, there may be site-specific or centralized costs within each phase or across cost categories; if relevant to analyses, these should be captured [28].

Follow-up within implementation studies is often short, even as one seeks to estimate longer-term cost (and potentially health) outcomes for downstream impacts. For example, decision-making about an intervention that prevents injurious falls, a significant source of morbidity and mortality for older adults, is best informed by estimates of costs saved per fall avoided. However, the impact of such an intervention may not be observed for months or years afterwards [47]. To overcome limited follow-up, simulation modeling can be used to explore the cost or health impacts of the intervention beyond short-term outcomes (and guide resource allocation decisions) [48–50]. The use of modeling also can highlight the spillover effects of the intervention to other conditions or sectors, including substitution of services, and allow analysts to perform sensitivity analyses. Implementation scientists need to account for any differential timing of costs if they occur beyond one year, by discounting costs (and benefits) using context-relevant discount rates (e.g., 3.5% for studies in UK settings [NICE]; 3% for studies in the USA) [16, 22, 51].

## Costs and economic analysis by analytic perspective

Depending on the stakeholders or decision-makers, there can be many analytic perspectives to consider for the cost and economic analysis of intervention implementation, for example, society (e.g., federal government program), patient, caregiver, healthcare system, healthcare sector, criminal justice system, employer, or public health department. The costs that should be included in an analysis are those that accrue to or are paid by the decision-maker, and in some cases, stakeholders may care about broader costs than ones attributed to them [52]. For example, a public health department may care about the implementation strategy and intervention’s costs associated with providing health services and the costs for participants, because if the cost burden is too high for the participants in the intervention, they may not participate, making the effort wasteful.

A societal perspective considers every potential cost associated with the implementation effort, including healthcare sector resources, non-healthcare sector resources, informal caregiver or volunteer time, patient time and transportation, costs of changes in patient productivity, and future costs related and unrelated to the intervention [16] (Fig. 1). Conducting an economic analysis with a long-run, societal perspective will identify cost-effective implementation strategies that maximize well-being for society, because it accounts for all economic impacts. Such broad evaluations may not provide information on the optimal policy from a specific perspective, however. A pragmatic solution for the implementation researcher would be to report on cost and economic analysis from multiple perspectives (e.g., health system, clinic, societal) and time horizons (e.g., 1 year, 5 years), which allows decision-makers to use the results relevant for them.

A recent study evaluated practice facilitation as an implementation strategy for improving small primary care practices’ adherence to clinical practice guidelines for cardiovascular risk reduction [53, 54]. To capture societal costs, one would include every potential cost, such as costs of practice facilitation, office staff time (e.g., meeting time, potentially office space), physician time and reimbursement for services, patient and caregiver time requirements stemming from primary care practice changes (e.g., smoking cessation counseling, lost productivity), immediate and downstream healthcare service use (e.g., smoking cessation medication, hospitalizations), and more. Estimated rent for required space that is above-and-beyond usual space use should be considered if space is scarce, because the space cannot be used for another purpose if it is used for the implementation strategy. The physician perspective, in deciding whether to participate, would include costs for physician time, supplies, and office staff time. The patient perspective would include costs of patient time, out-of-pocket costs, and potentially paid caregiver time, if patients pay for dependent care to participate in the intervention. If the local public health department were sponsoring the practice facilitator program, the public health department would want to know the costs of the implementation strategy (e.g., training and infrastructure to support practice facilitators, cost of their time in physician offices, travel time). A limited cost analysis was published, focusing on implementation strategy and practice staff time costs [54]. Such a narrow evaluation can lead to more questions, however, and leave other potential stakeholders who are interested in implementing such an approach wanting more information.

Another example of the potential implications of the choice of analytic perspective relates to a study of substance abuse treatment. Societal cost-effectiveness analyses have shown that substance abuse treatment is cost-effective [55]. Yet, the healthcare sector incurs the costs of substance use treatment, while the criminal justice sector reaps many of the benefits [55, 56], highlighting the possible conflict between decision-makers when costs and benefits accrue differentially to these sectors. A focused economic evaluation from the healthcare system perspective would make substance use treatment less appealing. This explains the paradoxical contraction in substance use treatment programs that has occurred over the past two decades [56].

Furthermore, broad economic evaluations that maximize social welfare may conflict with narrower, focused evaluations that are frequent in implementation science. Decision-makers often want a stylized analysis, such as a budget impact analysis [35, 57], or only care about the specific impact per patient. Focused evaluations that consider only specific categories of costs can lead to decisions that seem rational to the decision-maker, but are suboptimal for society and may not be social-welfare maximizing. Because an implementation strategy may affect evidence-based intervention utilization, efficiency, or downstream healthcare service use differentially, considering multiple cost categories is important; a narrowly defined implementation effort may in fact change how healthcare services are used in the near and longer term, which create a broader impact than is captured by only estimating implementation costs.

## Analysis and reporting of costs

As a rule, implementation scientists should sum the total costs of an implementation strategy and its impacts (and perhaps by stakeholder perspective) within an explicit time period (Table 1). This may be insufficient if such total costs vary across sites, however, because most decision-makers want to know what it would cost to adopt a strategy or program in their setting. Most costs will vary by some parameter (e.g., number of clinical staff, sites, participants) and over time. Implementation scientists will need to explore and report on such variation, so that decision-makers can anticipate or model the cost of the implementation strategy in new settings (or time periods).

In a study comparing group-based versus individual-based multidimensional-treatment foster care, the authors measured costs of feasibility, planning meetings, selection of service providers, staff training, fidelity monitoring and review, site visits, and feedback by stages of progress toward implementation [9]. By capturing subtotaled resources used (e.g., staff hours by job type, office space) and unit prices (e.g., wage rates, rent) and subtotaled implementation costs by week or month and by activity type (e.g., meetings, training), the analyst can conduct a more detailed analysis of costs. In particular, it would allow learning about such cost aspects as site-level variation in multi-site studies and whether costs change over time, potentially due to learning or efficiencies.

Ideally, implementation scientists should report both total intervention implementation costs and more granular cost measures. Because implementation strategies often target behavior change for organizations or professionals, the cost per practice or organization, per professional, or per end-user may be suitable metrics for the decision-maker. However, the major limitation of such denominators is the inability to attribute costs to a patient or participant and therefore to a subsequent health outcome; ultimately, the goal of implementing evidence-based interventions is to improve health outcomes and population health.

Sometimes a decision-maker wants to know how comparative implementation strategies with their evidence-based intervention affect health outcomes (Fig. 1). Such comparisons are facilitated by economic evaluations reporting the comparative costs by strategy with a measure of a patient’s health or well-being, such as life-years or quality-adjusted life-years gained. A denominator that is program and disease agnostic, such as quality-adjusted life year, allows for fair comparisons across strategies when resources are restricted.

Collecting information on patient outcomes may be burdensome and not central to an implementation study. Where there is robust evidence supporting an intervention being implemented, implementation studies may restrict themselves to measuring organizational and professional behavior change and report relevant outcomes, such as the additional cost for a percentage change in clinical guideline use. Other outcomes to consider for economic evaluation in implementation science could vary as widely as cost-per-patient or participant, cost-per-practice or per-health center, or cost-per-inappropriate MRI avoided. Implementation cost-effectiveness outcomes that incorporate patient-centered health outcomes can be added to decision analytic models by adapting the structure and evidence from intervention models [58].

To address the potential bias in estimating costs in implementation studies, sensitivity analyses are essential. Substituting job classifications, varying program capacity (i.e., estimate cost if the program is at full capacity versus initial rollout), reflecting regional or national wage rates, and estimating best/worst case are approaches to testing robustness of results.

## Summary

Implementation scientists should consider a broad range of cost categories when assessing the economic implications of strategies for implementing evidence-based care—and measure the costs relevant to the choice of analytic perspective(s), time horizon(s), and outcomes. Implementation scientists should explicitly consider costs from all sectors, such as healthcare, justice, and education, with strong consideration of the magnitude of costs for each sector and the cost or time burden of collecting the data. Although many implementation studies in healthcare focus exclusively on healthcare costs that are relevant for a particular decision-maker, a broader analysis may be useful or applicable to more than that decision-maker alone, leading one to consider additional, disaggregated, or divisible cost categories.

If researchers are using data that originate from accounting systems, where they can identify the variable and fixed costs, then they should determine how that system defines fixed costs and decide whether to treat those costs as fixed or variable given that the study time horizon may be different from the accounting system. Estimating costs by sector, phase, or even participant or patient subgroup can be useful to ensure a wide range of stakeholders understand the costs or savings that would accrue for their particular concern or perspective. Specific types of costs may affect adoption, implementation, fidelity, and sustainability.

Studies need to be explicit about each included cost category, type of cost data and its source, and how costs were calculated, to enhance transparency and improve adoption decisions by others. Engaging an expert in economic evaluation and key stakeholders prior to implementation can help inform which costs to capture and how. Making all analytic assumptions clear, justifiable, and unambiguous, e.g., durability of the intervention’s effect, time horizon, analytic perspective(s), and reasons for including/excluding specific costs, allows readers and decision-makers to replicate or compare results and apply them to their own settings. As outlined here, conducting analyses by societal, stakeholder, and sectoral perspectives will allow for broader uptake and relevance of value assessments by various decision-makers. Such value assessments can also help decision-makers reallocate resources to address health equity, such as redirecting resources to areas of greatest need [59].

## Data Availability

N/A
